# Effectiveness of a Multifaceted Informational-Based and Text Message Reminders on Pneumococcal and Influenza Vaccinations in Hospital Emergency Departments: A Cluster-Randomized Controlled Trial

**DOI:** 10.3390/vaccines9090962

**Published:** 2021-08-28

**Authors:** Sarah Tubiana, José Labarere, Jacques Levraut, Pierre Michelet, Fleur Jourda de Vaux, Benoit Doumenc, Pierre Hausfater, Christophe Choquet, Patrick Plaisance, Jeannot Schmidt, Véronique Mattei, Olivier Gacia, Didier Storme, Patrick Ray, Guillaume Der Sahakian, Marie-Clément Kouka, Laure Jainsky, Jocelyn Raude, Xavier Duval, Yann-Erick Claessens

**Affiliations:** 1 AP-HP, Hôpital Bichat, Centre d’Investigation Clinique, INSERM CIC 1425, F-75018 Paris, France; xavier.duval@aphp.fr; 2 Université de Paris, IAME, INSERM, F-75018 Paris, France; 3 Université de Grenoble Alpes/CNRS/TIMC UMR 5525, F-38041 Grenoble, France; JLabarere@chu-grenoble.fr; 4 Grenoble University Hospital, F-38043 Grenoble, France; 5 CHU de Nice, Département de Médecine d’Urgence, Hôpital Pasteur-II, F-06001 Nice, France; levraut.j@chu-nice.fr; 6 AP-HM, Hôpital la Timone, Département de Médecine d’Urgence, UMR C2VN-Aix Marseille Université, F-13005 Marseille, France; pierre.michelet@ap-hm.fr; 7 EMSP et Urgences Adultes-Hôpital Nord-APHM, F-13015 Marseille, France; fleur.jourda-de-vaux@ap-hm.fr; 8 AP-HP, Hôpital Cochin, Services d’Accueil des Urgences, F-75014 Paris, France; benoit.doumenc@aphp.fr; 9 AP-HP, Hôpital Pitié-Salpêtrière, Services d’Accueil des Urgences, GRC-14 BIOSFAST Sorbonne Université, F-75651 Paris, France; pierre.hausfater@aphp.fr; 10 AP-HP, Hôpital Bichat, Services d’Accueil des Urgences, F-75018 Paris, France; Christophe.choquet@aphp.fr; 11 AP-HP, Hôpital Lariboisière, Services d’Accueil des Urgences, F-75010 Paris, France; patrick.plaisance@aphp.fr; 12 CHU de Clermont-Ferrand, Services d’Accueil des Urgences, F-63003 Clermont-Ferrand, France; jschmidt@chu-clermontferrand.fr; 13 CH de Menton, Services d’Accueil des Urgences, F-06507 Menton, France; v.mattei@ch-menton.fr; 14 SSA, HIA Laveran, Services d’Accueil des Urgences, F-13384 Marseille, France; olivier.gacia@intradef.gouv.fr; 15 CH Jacques Lacarin, Services d’Accueil des Urgences, F-03207 Vichy, France; didier.storme@ch-vichy.fr; 16 CHU Mitterrand, Département Universitaire de Médecine d’Urgence, Université de Bourgogne, F-21000 Dijon, France; patrick.ray@chu-dijon.fr; 17 Centre Hospitalier d’Orange, FMI Nord Vaucluse, F-84104 Orange, France; dersahakian@hotmail.fr; 18 Hôpital Delafontaine, Services d’Accueil des Urgences, F-93200 Saint Denis, France; marie-clement.kouka@ch-stdenis.fr; 19 CH Paul Ardier Issoire, Services d’Accueil des Urgences, F-63500 Issoire, France; ljainsky@ch-issoire.fr; 20 EHESP-School of Public Health, F-35043 Rennes, France; Jocelyn.Raude@ehesp.fr; 21 Hôpital Princesse Grâce, Service d’accueil des Urgences, MC-98012 Monaco, Monaco; yann-erick.claessens@chpg.mc

**Keywords:** pneumococcal vaccine, influenza vaccine, reminder systems, emergency departments, cluster-randomized trial

## Abstract

Objectives. We aimed to evaluate the effectiveness of a multifaceted procedure in improving pneumococcal and influenza vaccinations 6 months after an emergency department (ED) visit among patients aged 65 years and older. Methods. We conducted a cluster-randomized, controlled, parallel-group, open-label implementation trial in 18 EDs in France and Monaco. Participants were recruited from November 2015 to September 2016. EDs were randomly assigned with a 1:1 ratio to provide either a multifaceted procedure that combined structured information about pneumococcal and influenza vaccines and three text message reminders sent to patients every two weeks (intervention arm) or nonstructured information only (control arm). The outcomes were self-reported pneumococcal vaccination and influenza vaccination rates within 6 months of enrollment. Results. A total of 9 EDs were randomized to the intervention arm (n = 780 patients) and 9 to the control arm (n = 695 patients). The median age for all enrolled patients was 74 years (25–75th percentiles, 69 to 82): 50.1% were male, 34.9% had at least one underlying condition, and 30.7% were at risk for invasive pneumococcal infection. In the intention-to-treat analysis, the multifaceted intervention did not alter the pneumococcal vaccination rate (6.4% versus 4.6%, absolute difference: 1.8; 95% CI: [−0.9 to 4.4]; *p* = 0.19), whereas it improved the influenza vaccination rate (52.1% versus 40.0%, absolute difference: 12.1; 95% CI: [2.4 to 21.8]; *p* = 0.01). At 12 months, mortality did not differ between the intervention (9.7%) and control (11.2%) arms (*p* = 0.35). Conclusions. A multifaceted intervention based on text message reminders provides an opportunity to increase anti-influenza vaccination among elderly patients visiting the ED. Efforts are warranted to provide better information on pneumococcal diseases and the benefits of pneumococcal vaccines, especially in the elderly.

## 1. Introduction

Community-acquired pneumonia and seasonal influenza are major causes of morbimortality worldwide, especially among the elderly and individuals with chronic underlying conditions. Influenza and pneumococcal vaccines have been shown to be effective in reducing the risk of influenza occurrence and severity and *Streptococcus pneumoniae* infections, respectively, in elderly people [1,2,3]. Current international guidelines recommend that all people aged 65 years or older receive influenza vaccination every year [4] and pneumococcal vaccination with single doses of PCV13 and PPV23 [5]. In France, seasonal influenza vaccination is recommended annually for people older than 65 years and for younger individuals with chronic underlying diseases. Pneumococcal vaccination is recommended on its part for people at risk of invasive pneumococcal infection, i.e., immunocompromised and nonimmunocompromised individuals with an underlying condition, irrespective of their age.

Despite the efforts of primary care providers and public health agencies, vaccine coverage has remained far below the expected coverage rate [6,7]. The most common barriers to vaccination include a perceived lack of safety, a deficit of awareness, complacency (i.e., doubts about the necessity of vaccines), and vaccine misconceptions [8]. To address the growing vaccine hesitancy, a variety of strategies have been implemented and evaluated across countries [9]. Most of them are related to the COM-B model for adherence to health recommendations, which assumes that people’s capacities, motivations, and opportunities are fundamental factors in the engagement of health-protective behavior [10]. Among the most promising interventions based on the COM-B model, those using external cues to action (i.e., circumstances that trigger the decision-making process leading to the uptake of the recommended vaccine, such as advice from a healthcare worker or illness of a friend/relative) seem to be generally effective to address vaccine hesitancy [11]. They include interventions using text messaging systems to promote vaccination in local healthcare facilities and training healthcare providers to improve their vaccine recommendations [12,13].

Every year, one-third of the population visits hospital emergency departments (EDs) in France. Overall, 10–20% of the adult population makes at least one visit to the ED annually in the United States [14]. Regardless of the reason for the visit, EDs are a location to assess the general health status of the population; furthermore, a visit to the ED may be an opportunity to initiate interventions to improve patient’s adherence to vaccine recommendations.

The aim of this study was to determine whether a multifaceted intervention providing patients aged 65 years and older with structured information at the time of ED visit and 3 text message reminders increased pneumococcal and influenza vaccination rates at 6 months of follow-up.

## 2. Methods

### 2.1. Study Design and Setting

The IMPROVED study is a cluster-randomized, controlled, parallel-group, open-label implementation trial conducted in the ED of 18 acute care hospitals in France and Monaco. The rationale and methods for the IMPROVED trial were prespecified and reported in a protocol registered at ClinicalTrials.gov (NCT01899365).

### 2.2. Participants

Consecutive patients aged 65 years and older attending EDs from the community or nursing home setting were screened for eligibility on weekdays between November 2015 and September 2016. Participation in the study was proposed after management of the reason for the ED visit was initiated. Participants were enrolled regardless of the reason for ED visit. Exclusion criteria included refusal to participate, inability to receive mobile phone text messages, dementia, altered mental status, language restriction, previous pneumococcal vaccination, or contraindication to pneumococcal vaccination.

### 2.3. Randomization

Random allocation was performed at the hospital level in order to prevent unintentional spill-over of intervention effects from one study arm to another [15]. A statistician generated a random allocation sequence, with a 1:1 ratio, and hospitals were matched by university affiliation status, annual number of ED admissions (<40,000 versus ≥40,000), and areas (Paris versus other regions) in order to balance recruitment between study arms. Because the study arm was released at the beginning of the enrollment period, we could not ensure allocation concealment.

### 2.4. Study Intervention

Patients randomized in the experimental arm received the multifaceted intervention procedure at the end of the ED visit. It comprised a brief structured interview with the physician about pneumococcal and influenza burden and the interest of both vaccinations. An information sheet with an explanation about the risks and benefits of vaccination and a letter dedicated to the general practitioner stating that the patient was at risk for pneumococcal infection and could benefit from pneumococcal vaccination were given to the patients. Three text messages were sent to patients every 2 weeks to remind them to talk about pneumococcal risk with their general practitioners.

Patients randomized in the control arm received a brief nonstructured interview with the physician about pneumococcal risk and vaccination, an information sheet about the risks and benefits of vaccination, and a letter for their general practitioner stating that the patient was at risk for pneumococcal infection and could benefit from pneumococcal vaccination.

### 2.5. Blinding

In this open-label trial, patients, care providers, investigators, and outcome assessors were not blinded to allocation. Only the statistician was blinded to study arm and hospital until all analyses planned in the statistical analysis plan (SAP) were performed.

### 2.6. Data Collection

Using an electronic case report form, ED physicians prospectively recorded information on baseline patient characteristics, including demographics, reason for ED visit, comorbid conditions, prior history of pneumonia, meningitis or chronic sinusitis, and influenza vaccination status. Patients filled out a questionnaire during the ED visit and were contacted at 6 and 12 months following enrollment for a structured telephone interview with a study clinical research assistant.

### 2.7. Outcomes

The primary outcome was self-reported pneumococcal vaccination within 6 months of enrollment. Secondary outcomes included 6-month self-reported influenza vaccination and 12-month all-cause mortality. All-cause mortality and date of death were captured by requesting the National Death Index in France (CepiDC) and the city council (DASS) in Monaco.

### 2.8. Sample Size

Assuming a 0.05 intra-cluster correlation coefficient, we estimated that 1800 patients (i.e., 900 patients per study arm) would be required to achieve 80% power for detecting an absolute difference of 15 percentage points in the 6-month pneumococcal vaccination rate between the intervention (35%) and control (20%) study arms in the intention-to-treat analysis, with a two-sided alpha level set at 0.05. Anticipating an average number of 100 patients per ED, 18 study sites were required. Because of noncompliance with the recruitment plan, the sponsor and the steering committee decided to stop recruitment on 5 July 2017.

### 2.9. Statistical Analysis

The intention-to-treat population consisted of all enrolled patients recorded in the database, with the exception of those who withdrew informed consent. All enrolled patients were analyzed in the study arm to which they were randomly allocated regardless of whether they received the assigned intervention and irrespective of any protocol deviations or violations.

Descriptive summary statistics were used for reporting continuous (median and 25th–75th percentiles) and categorical (numbers and percentages) variables. We derived odds ratios and absolute difference in 6-month pneumococcal vaccination between study arm point estimates along with the 95% confidence interval (CI) from the logistic regression for the dependent binary variable with the study arm entered as the independent variable. To account for patient clustering within the ED, we used the random intercept model. We performed multiple imputations of missing values for the pneumococcal vaccination using multivariate regression with an iterative Markov chain Monte Carlo (MCMC) method. Fifty imputed data sets were created with a total run length of 50,000 iterations, and imputations were made every 1000 iterations. Variables included in the imputation model are listed in Supplementary (Table S1).

To assess the robustness of our findings, we repeated the primary outcome analysis after discarding patients with a missing value for the primary outcome (i.e., casewise analysis) and after restricting the analytical sample to patients who were at risk for invasive pneumococcal infection, i.e., immunocompromised and nonimmunocompromised individuals with an underlying condition for whom pneumococcal vaccination was recommended in France (referred as ‘at-risk patients’ analysis). The same approach was applied to compare influenza vaccination and 6- and 12-month all-cause mortality between study arms.

We performed multivariable analysis to assess the effect of imbalances in baseline characteristics between study groups on self-reported 6-month pneumococcal and influenza vaccinations. Odds ratios were adjusted for baseline ED (location in Paris and suburbs, university affiliation, annual volume ≥40,000) and patient (age, gender, enrollment between 1 November and 30 April, reason for ED visit, underlying conditions, history of pneumonia, history of chronic sinusitis, history of meningitis, institution dwelling, history of influenza vaccination, and hospital admission) characteristics.

Two-tailed values of *p* > 0.05 were considered statistically significant. All statistical analyses were conducted using Stata 14.0 Special Edition (StataCorp, College Station, TX, USA).

### 2.10. Ethics and Regulatory Issues

This study complied with the Declaration of Helsinki for research involving human subjects. An institutional review board (Comité de Protection des Personnes Ile de France 1, IRB 00008522, 2014-janvier-13467), the French Data Protection Authority (Commission nationale de l’informatique et des libertés) (DR-2015-108), and ANSM (IDRCB: 2013-A00943-42) reviewed and approved the study protocol and the consent form prior to study initiation. All participants provided informed consent.

## 3. Results

### 3.1. Study Population

**Figure 1:**
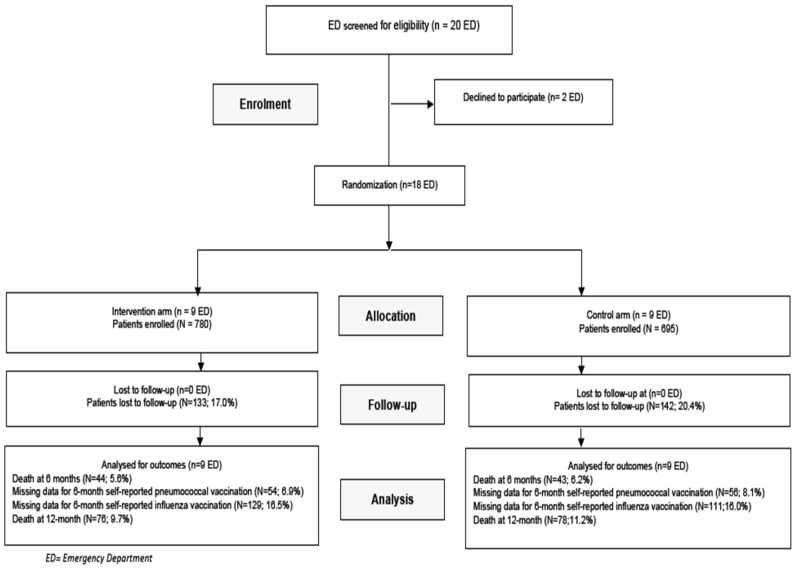
Consort flow diagram, IMPROVED trial. ED = emergency department.

Of 20 emergency departments invited to participate, 2 declined, and 18 were randomized (9 in the experimental arm and 9 in the control arm, Figure 1). Between 9 November 2015 and 5 July 2017, 1475 patients (780 in the intervention arm and 695 in the control arm) were enrolled (Figure 1). The median number of patients enrolled in each emergency department was 98 (range: 18 to 145) (Table S2).

The median age for all enrolled patients was 74 years (25–75th percentiles, 69 to 82): 50.1% were male, 34.9% had at least one underlying condition, and 30.7% were at risk for invasive pneumococcal infection (Table 1). The two study arms were well balanced with respect to baseline characteristics, with the exception of age, reasons for ED visit, history of chronic sinusitis, and chronic respiratory diseases. Patients assigned to the intervention arm were less likely to be enrolled between 1 November and 30 April (Figure S1).

### 3.2. Self-Reported Pneumococcal Vaccination

Information on self-reported pneumococcal vaccination was available for 1003 patients (549 (70.3%) in the intervention arm and 454 (65.3%) in the control arm; *p* = 0.04). Missing values for the primary outcome were imputed for 87 patients who died within 6 months (44 (5.6%) in the intervention arm and 43 (6.2%) in the control arm; *p* = 0.66), 275 who were lost to follow-up at 6 months (133 (17.0%) in the intervention arm and 142 (20.4%) in the control arm; *p* = 0.10), and 110 who did not answer (54 6.9%) in the intervention arm and 56 8.1%) in the control arm; *p* = 0.14) (Table 2). The estimated percentages of pneumococcal vaccination were 6.4% and 4.6% in the intervention and control arms, respectively (absolute difference: 1.8 percentage points; 95% CI: −0.9 to 4.4; *p* = 0.19) (Table 3). Results from casewise and ‘at-risk patients’ analyses were rather similar (Tables S3 and S4). In univariable analysis, baseline characteristics associated with self-reported pneumococcal vaccination included ED visit for respiratory tract infection, underlying immunosuppression, history of chronic sinusitis, and recent history of influenza vaccination (Table S5). After adjusting for baseline characteristics, no significant association was observed between the intervention arm and self-reported pneumococcal vaccination (Table S6).

### 3.3. Self-Reported Influenza Vaccination

The secondary outcome of self-reported influenza vaccination was available in 873 patients (474 60.8%) in the intervention arm and 399 (57.4%)) in the control arm; *p* = 0.19) (Table 2). The estimated percentages of influenza vaccination were 52.1% and 40.0% in the intervention and control arms, respectively (absolute difference: 12.1 percentage points; 95% CI: 2.4 to 21.8; *p* = 0.01) (Table 3). Estimates were unchanged in casewise analysis (Table S3)). In univariable analysis, baseline characteristics associated with self-reported influenza vaccination included advancing age, female gender, history of stroke, and previous history of influenza vaccination (Table S7). After adjusting for baseline characteristics, the intervention arm remained significantly associated with an increased odds ratio of self-reported influenza vaccination (Table S6).

### 3.4. 6-Month and 12-Month All-Cause Mortality

All-cause mortality did not differ between the intervention and control arms at 6 months (absolute difference: −0.5 percentage points: 95% CI: −3.0 to 1.9; *p* = 0.66) and 12 months of enrollment (absolute difference: −1.4 percentage points; 95% CI: −6.1 to 3.2; *p* = 0.54) (Table 3).

## 4. Discussion

In this cluster-randomized, controlled, parallel-group, open-label, implementation trial, provision of information during ED visit followed by reminders through text messages did not alter 6-month pneumococcal vaccination but was associated with significant improvement in influenza vaccination rate among patients aged 65 years and older.

To enhance the generalizability of our results, we enrolled consecutive patients attending EDs during the daytime, thanks to dedicated research staff in each study site. All patients aged 65 years and older were eligible to participate, regardless of the reason for admission to ED, except those already vaccinated against *S. pneumoniae*. We enrolled patients visiting hospitals located in different areas in France and Monaco with a large population pool in both tertiary and nontertiary centers. Our study population was representative of elderly patients presenting to the ED in France, with regard to medical reasons for ED visit and subsequent hospital admission rate [16]. At baseline, 50.1% of participants reported influenza vaccination coverage for the current season, which was close to the French national estimates ranging from 46% to 48% during the 2015–2017 period, for people older than 65 years old [17]. These observations make us believe that the results of our trial can be extrapolated to other French EDs.

Current international guidelines recommend that individuals aged 65 years or older receive annual influenza vaccination and pneumococcal vaccination every five years [3,5]. In France, this is also the case for seasonal influenza vaccination, whereas pneumococcal vaccination is restricted to people at risk for invasive pneumococcal infection. Although age above 65 years is not per se considered as an indication for pneumococcal vaccination in France, pneumococcal vaccines had a marketing authorization for all adults above 65 years of age because the overall incidence of pneumococcal disease rises greatly in this age group [18]. Results of the analysis restricted to the ‘at-risk’ subgroup for which pneumococcal vaccination is recommended were consistent with those for the overall study population.

Our study was conducted in the setting of the ED. There are on average 21 million ED visits per year over the last decade [16]. Of those, around 20% involve adults aged 75 or more [19]. Previous studies have reported that up to 69% and 45% of patients visiting the ED were at high risk for influenza or pneumococcal disease, respectively, although less than 20% of that high-risk population were vaccinated [14]. It should be noted that patients usually visit the ED for acute medical conditions requiring curative care and can be reluctant to vaccinate in the ED, as this is not the main reason for ED visit. Patients refer to their general practitioner for health prevention. To note, emergency physicians daily vaccinate many patients against tetanus but do not offer influenza and pneumococcal vaccinations [20]. However, they can easily enroll patients in intervention programs that provide reminders and identify local sources of vaccines, without increased staffing.

A recent cluster-randomized crossover trial, conducted in primary care in Singapore, showed that point-of-care informational interventions (i.e., flyers and posters) contributed to increased influenza and pneumococcal vaccination uptake [21]. Numerous studies showed that patient reminder and recall systems improved immunization rates in various types of settings, based on moderate certainty evidence [22], but few of them focused on text message interventions. Herrett et al. [23] and Regan et al. [24] found a modest improvement in influenza vaccine uptake among at-risk groups in the United Kingdom and Australia. In the USA, a patient-level randomized controlled trial was implemented in a low-income population and showed that text messages sent to parents were associated with a 9% relative increase in influenza vaccine uptake among children [13]. Although people across all age groups communicate via text messaging, further research is still needed to investigate the effectiveness of this mode of communication on vaccination uptake among seniors [11].

Our multifaceted intervention procedure based on external cues to action combined structured oral interviews, written information, and text messages as reminders. Although our study intervention combined different components that were more resource and time consuming than text message reminders, we did not find evidence of effectiveness on pneumococcal vaccination uptake. There are several potential explanations for these negative findings. First, our intervention might have been hampered by the tension in the supply of pneumococcal vaccines (PPV23), leading even to the point of stock-outs in pharmacies at the end of 2017, in France. Second, our intervention might have been attenuated by French guidelines advocating pneumococcal vaccination for at-risk patients but our findings were rather comparable in the ‘at-risk’ population.

Interestingly, our multifaceted intervention significantly increased influenza vaccination uptake whereas half of the participants reported being previously vaccinated against influenza. We previously found that history of seasonal influenza vaccination was associated with willingness to be vaccinated [25]. Participants in our study were not previously vaccinated against pneumococcal and therefore might be less prone to vaccination. Second, awareness of the pneumococcal vaccination recommendation is probably lower among both physicians and patients than for influenza vaccine [26]. Seasonal influenza vaccination is well publicized across all age groups promoted by campaigns and health professionals and in workplaces, whereas pneumococcal vaccination appears to have a much lower promotion [27]. Patients aged 65 years and older receive a voucher from the French national health insurance to be vaccinated against influenza, whereas this is not the case for pneumococcus vaccine. Finally, the content of the message contributes to the success of interventions. It is possible that there may have been a misunderstanding between influenza infection and the risk-related pulmonary complications and pneumococcal infection itself.

We acknowledge several limitations to our study. First, vaccine uptake was self-reported and not verified by vaccination booklets or medical records. Second, no data were available on the proportion of patients who effectively received or read the text messages. Third, an imbalance was observed in baseline patient characteristics in this cluster-randomized trial. Although the results were unchanged in multivariable analysis, we cannot exclude residual confounding by an imbalance in unmeasured characteristics. Fourth, heterogeneity in participation rates across study sites could not be investigated because this information was not recorded in this trial. Fifth, external factors may have hampered pneumococcal vaccination, such as the tension in the supply of pneumococcal vaccines in France. Sixth, because of noncompliance with the recruitment plan, the study had to be stopped prematurely before reaching the required number of subjects.

To conclude, a multifaceted intervention delivered in the ED increased influenza vaccination coverage but had no significant effect on pneumococcal vaccination coverage among elderly patients 6 months after ED visit. Whatever the reason for visiting hospital EDs, it can be an opportunity to educate patients about the importance of vaccinations and to implement automated reminder systems. However, the heterogeneity in effectiveness between the two vaccinations shows the complexity of receiving this message when it comes to diseases that receive little media coverage and are therefore not well known by patients. Efforts are warranted to provide better information on pneumococcal diseases and the benefits of antipneumococcal vaccine, especially in the elderly.

## Figures and Tables

**Table 1:** Comparison of baseline patient characteristics between study arms; IMPROVED trial.

Baseline Characteristics	All (n = 1475) n or med (% or IQR)		Intervention (n = 780) n or med (% or IQR)		Control (n = 695) n or med (% or IQR)		*p*-Value
Age (year)	74	(69–82)	74	(68–80)	76	(69–83)	<0.001
Male gender	739	(50.1)	389	(49.9)	350	(50.4)	0.85
**Study site**							
Location in Paris and suburbs	517	(35.1)	300	(38.5)	217	(31.2)	0.004
University hospital	874	(59.3)	499	(64.0)	375	(54.0)	<0.001
ED annual volume ≥ 40,000	916	(62.1)	499	(64.0)	417	(60.0)	0.12
Enrolled between Nov. and Apr.	865	(58.6)	371	(47.6)	494	(71.1)	<0.001
**Reason for ED visit**							0.002
Respiratory tract infection	93	(6.3)	41	(5.3)	52	(7.5)	
Nonrespiratory tract infection	42	(2.8)	20	(2.6)	22	(3.2)	
Medical reason	934	(63.3)	525	(67.3)	409	(58.8)	
Traumatic reason	291	(19.7)	151	(19.4)	140	(20.1)	
Nontraumatic surgery	23	(1.6)	9	(1.1)	14	(2.0)	
Psychiatry	7	(0.5)	4	(0.5)	3	(0.4)	
Other	85	(5.8)	30	(3.8)	55	(7.9)	
**Medical history**							
At least one underlying condition	515	(34.9)	271	(34.7)	244	(35.1)	0.88
Chronic respiratory disease	96	(6.5)	37	(4.7)	59	(8.5)	0.004
Heart failure	209	(14.2)	119	(15.3)	90	(12.9)	0.20
Immunosuppression	17	(1.1)	7	(0.9)	10	(1.4)	0.33
History of stroke	97	(6.6)	48	(6.1)	49	(7.0)	0.49
Chronic renal failure	70	(4.7)	30	(3.8)	40	(5.8)	0.08
Cirrhosis	17	(1.1)	10	(1.3)	7	(1.0)	0.62
Cancer	137	(9.3)	83	(10.6)	54	(7.8)	0.06
History of pneumonia	87	(5.9)	47	(6.0)	40	(5.8)	0.83
History of meningitis	9	(0.6)	5	(0.6)	4	(0.6)	>0.999
History of chronic sinusitis	41	(2.8)	31	(4.0)	10	(1.4)	0.003
Institution dwelling	19	(1.3)	10	(1.3)	9	(1.3)	>0.999
**History of influenza vaccination**							0.26
Current season influenza vaccination	739	(50.1)	387	(49.6)	352	(50.6)	
influenza vaccination > 1 year	240	(16.3)	134	(17.2)	106	(15.2)	
No history of influenza vaccination	493	(33.4)	259	(33.2)	234	(33.7)	
Missing data	3	(0.2)	0	(0.0)	3	(0.4)	
At risk of invasive pneumococcal infection *	453	(30.7)	241	(30.9)	212	(30.5)	0.87
**Hospitalization**							0.91
Yes	766	(51.9)	409	(52.4)	357	(51.4)	
No	702	(47.6)	367	(47.0)	335	(48.2)	
Missing data	7	(0.5)	4	(0.5)	3	(0.4)	

Abbreviations: ED = emergency department. * including immune suppression, sickle cell disease, active cancer, congestive heart failure, chronic respiratory disease, renal disease, cirrhosis, and history of meningitis.

**Table 2:** Comparison of 6-month and 12-month outcomes between study arms; IMPROVED trial.

Outcomes	All (n = 1475) n (%)		Intervention (n = 780) n (%)		Control (n = 695) n (%)		*p*-Value
**6-month outcomes**							
Lost to follow-up	275	(18.6)	133	(17.0)	142	(20.4)	0.10
Death	87	(5.9)	44	(5.6)	43	(6.2)	0.66
Self-reported pneumococcal vaccination							0.14
Yes	62	(4.2)	38	(4.9)	24	(3.4)	
No	941	(63.8)	511	(65.5)	430	(61.9)	
No response	110	(7.5)	54	(6.9)	56	(8.1)	
Not specified (death or lost to follow-up)	362	(24.5)	177	(22.7)	185	(26.6)	
Self-reported influenza vaccination							<0.001
Yes	387	(26.2)	239	(30.6)	148	(21.3)	
No	486	(32.9)	235	(30.1)	251	(36.1)	
No response	240	(16.3)	129	(16.5)	111	(16.0)	
Not specified (death or lost to follow-up)	362	(24.5)	177	(22.7)	185	(26.6)	
**12-month outcome**							
Death	154	(10.4)	76	(9.7)	78	(11.2)	0.35

**Table 3:** Comparison of primary and secondary outcomes in intention-to-treat analysis; IMPROVED trial (N = 1475).

Outcome	Intervention (n = 780)	Control (n = 695)	Odds Ratio * (95% CI)		Absolute Difference * (95% CI)		*p*-Value
Self-reported 6-month pneumococcal vaccination; %	6.4	4.6	1.41	(0.84 to 2.37)	1.8	(−0.9 to 4.4)	0.19
Self-reported 6-month influenza vaccination, %	52.1	40.0	1.63	(1.10 to 2.42)	12.1	(2.4 to 21.8)	0.01
6-month all-cause mortality, %	5.6	6.2	0.91	(0.59 to 1.40)	−0.5	(−3.0 to 1.9)	0.66
12-month all-cause mortality, %	9.0	10.5	0.85	(0.50 to 1.43)	−1.4	(−6.1 to 3.2)	0.54

Abbreviations: CI = confidence interval. * Odds ratio and absolute difference point estimates along with 95% confidence intervals were derived from logistic regression for the dependent binary variable with the study arm entered as the independent variable. To account for patient clustering within ED, we used a random intercept model. Missing values for self-reported pneumococcal and influenza vaccination were replaced by multiple imputation.

## Supplementary Materials

The following are available online at https://www.mdpi.com/article/10.3390/vaccines9090962/s1, Figure S1: Numbers of patients enrolled per month in the intervention and control groups, IMPROVED trial, Table S1: Variables entered in the multivariate imputation model, IMPROVED trial, Table S2: Characteristics of Emergency Departments participating in the IMPROVED trial, Table S3: Comparison of primary and secondary outcomes in casewise analysis; IMPROVED trial, Table S4: Comparison of primary and secondary outcomes restricted to the subgroup of ‘at risk’ patients in intention-to-treat analysis; IMPROVED trial, Table S5: Comparison of baseline characteristics according to self-reported 6-month pneumococcal vaccination; IMPROVED trial, Table S6: Adjusted odds ratio of self-reported pneumococcal and influenza vaccination associated with intervention arm in intention-to-treat analysis; IMPROVED trial (N = 1475), Table S7: Comparison of baseline patient characteristics according to self-reported 6-month influenza vaccination; IMPROVED trial.
