# Supplementary material for: Effectiveness of a Multifaceted Informational-Based and Text Message Reminders on Pneumococcal and Influenza Vaccinations in Hospital Emergency Departments: A Cluster-Randomized Controlled Trial
Source: Vaccines (Basel). 2021 Aug 28;9(9):962. doi: 10.3390/vaccines9090962 (PMC8473355; doi:10.3390/vaccines9090962)
Supplement: Supplementary file 1 [file vaccines-09-00962-s001.zip › vaccines-1310616-supplementary.pdf]

**Supplementary Material**

Figure S1. Numbers of patients enrolled per month in the intervention and control groups, IMPROVED trial.

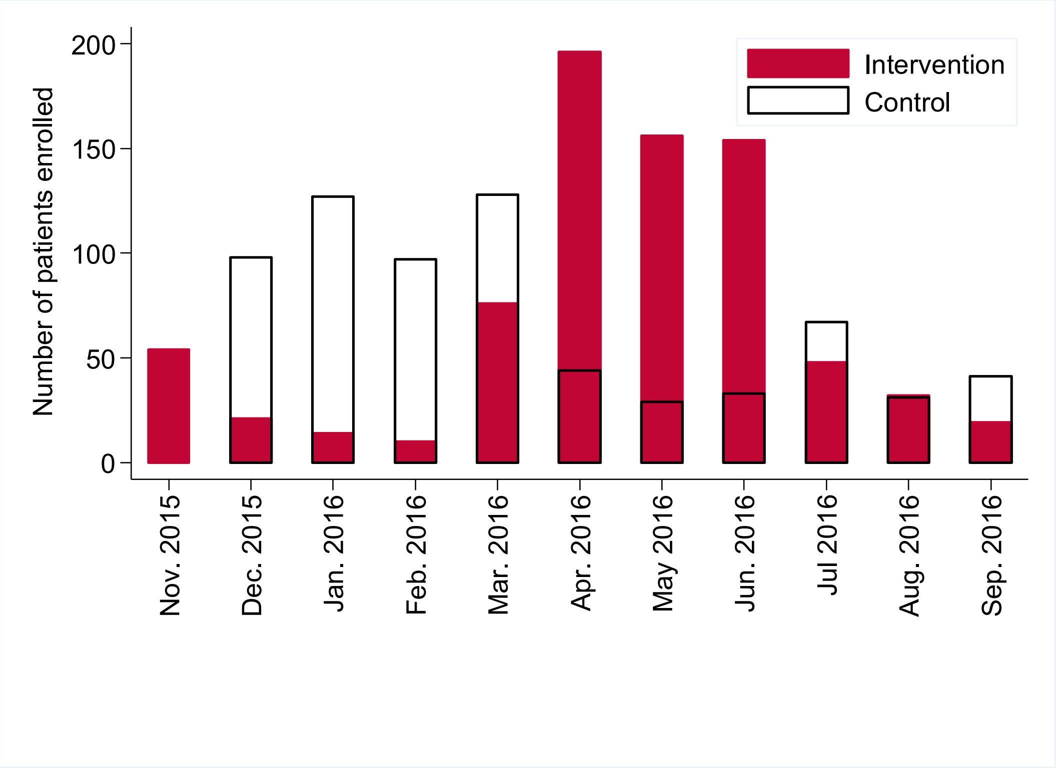

Table S1. Variables entered in the multivariate imputation model, IMPROVED trial.

| | Number of Observations per Imputation Set | | | |
|---|---|---|---|---|
| Variable | Complete | Incomplete | Imputed | Total |
| 6-month pneumococcal vaccination status | 1003 | 472 | 472 | 1475 |
| 6-month influenza vaccination status | 873 | 602 | 602 | 1475 |
| History of influenza vaccination | 1472 | 3 | 3 | 1475 |
| Hospital admission | 1468 | 7 | 7 | 1475 |
| Study site | 1475 | 0 | 0 | 1475 |
| Age | 1475 | 0 | 0 | 1475 |
| Gender | 1475 | 0 | 0 | 1475 |
| ED visit diagnosis | 1475 | 0 | 0 | 1475 |
| Chronic respiratory disease | 1475 | 0 | 0 | 1475 |
| Congestive heart failure | 1475 | 0 | 0 | 1475 |
| History of stroke | 1475 | 0 | 0 | 1475 |
| Immunosuppression | 1475 | 0 | 0 | 1475 |
| Chronic renal failure | 1475 | 0 | 0 | 1475 |
| Cirrhosis | 1475 | 0 | 0 | 1475 |
| Cancer | 1475 | 0 | 0 | 1475 |
| History of pneumonia | 1475 | 0 | 0 | 1475 |
| History of chronic sinusitis | 1475 | 0 | 0 | 1475 |
| History of meningitis | 1475 | 0 | 0 | 1475 |
| Institution dwelling | 1475 | 0 | 0 | 1475 |

Table S2. Characteristics of Emergency Departments participating in the IMPROVED trial

| Emergency Departments Characteristics | All (*n*=18) | | Intervention (*n*=9) | | Control (*n*=9) | |
|---|---|---|---|---|---|---|
| University-affiliated hospitals, *n* | 9 | | 5 | | 4 | |
| Annual ED visits ≥40,000, *n* | 10 | | 5 | | 5 | |
| Paris and suburbs, *n* | 6 | | 3 | | 3 | |
| Number of patients included, median (range) | 98 | (18–145) | 100 | (18–145) | 88 | (24–100) |

Table S3. Comparison of primary and secondary outcomes in casewise analysis; IMPROVED trial

| Outcome | Intervention, n/N (%) | | Control, n/N (%) | | Odds Ratio (95% CI) | | Absolute Difference (95% CI) | | *P-Value* |
|---|---|---|---|---|---|---|---|---|---|
| Self-reported 6-month pneumococcal vaccination | 38/549 | (6.9) | 24/454 | (5.3) | 1.33 | (0.79 to 2.26) | 1.6 | (−1.3 to 4.6) | 0.28 |
| Self-reported 6-month influenza vaccination | 239/474 | (50.4) | 148/399 | (37.1) | 1.91 | (1.12 to 3.25) | 15.9 | (3.0 to 28.8) | <0.001 |

*Abbreviations: CI = confidence interval.*

** Odds ratio and absolute difference point estimates along with 95% confidence intervals were derived from logistic regression for dependent binary variable with study arm entered as independent variable. To account for patient clustering within ED, we used random intercept model.*

Table S4. Comparison of primary and secondary outcomes restricted to the subgroup of 'at risk' patients in intention-to-treat analysis; IMPROVED trial

| Outcome | Intervention (n = 241) | Control (n = 212) | Odds Ratio * (95% CI) | | Absolute Difference * (95% CI) | | *P-Value* |
|---|---|---|---|---|---|---|---|
| Self-reported 6-month pneumococcal vaccination, % | 7.5 | 7.0 | 1.08 | (0.48 to 2.43) | 0.5 | (−4.8 to 5.9) | 0.85 |
| Self-reported 6-month influenza vaccination, % | 49.0 | 43.8 | 1.23 | (0.75 to 2.01) | 5.2 | (−7.0 to 17.3) | 0.41 |
| 6-month all-cause mortality, % | 13.3 | 10.9 | 1.26 | (0.71 to 2.23) | 2.4 | (−3.6 to 8.4) | 0.43 |
| 12-month all-cause mortality, % | 20.7 | 20.3 | 1.02 | (0.58 to 1.79) | 0.4 | (−8.7 to 9.5) | 0.94 |

*Abbreviations: CI = confidence interval.*

** Odds ratio and absolute difference point estimates along with 95% confidence intervals were derived from logistic regression for dependent binary variable with study arm entered as independent variable. To account for patient clustering within ED, we used random intercept model. Missing values for self-reported pneumococcal and influenza vaccination were replaced by multiple imputation.*

Table S5. Comparison of baseline characteristics according to self-reported 6-month pneumococcal vaccination; IMPROVED trial.

| | Self-Reported 6-Month Pneumococcal Vaccination * | | | | |
|---|---|---|---|---|---|
| | No (*n* = 941) n or med (% or IQR) | | Yes (*n* = 62) n or med (% or IQR) | | *P-Value* |
| Age (year) | 74 | (69–81) | 75 | (69–81) | 0.46 |
| Gender (female) | 460 | (48.9) | 33 | (53.2) | 0.51 |
| Study site | | | | | |
| Location in Paris and suburbs | 314 | (33.4) | 21 | (33.9) | 0.94 |
| University hospital | 516 | (54.8) | 41 | (66.1) | 0.08 |
| ED annual volume ≥40,000 | 546 | (58.0) | 41 | (66.1) | 0.21 |
| Enrollment between November and April | 564 | (59.9) | 29 | (46.8) | 0.04 |
| Reason for ED visit | | | | | 0.004 |
| Respiratory tract infection | 44 | (4.7) | 11 | (17.7) | |
| Non-respiratory tract infection | 21 | (2.2) | 2 | (3.2) | |
| Medical reason | 595 | (63.2) | 39 | (62.9) | |
| Traumatic reason | 203 | (21.6) | 8 | (12.9) | |
| Non-traumatic surgery | 14 | (1.5) | 1 | (1.6) | |
| Psychiatry | 3 | (0.3) | 0 | (0.0) | |
| Other | 61 | (6.5) | 1 | (1.6) | |
| At least one underlying condition | 265 | (28.2) | 24 | (38.7) | 0.08 |
| Chronic respiratory disease | 48 | (5.1) | 6 | (9.7) | 0.14 |
| Heart failure | 96 | (10.2) | 11 | (17.7) | 0.06 |
| Immunosuppression | 6 | (0.6) | 4 | (6.5) | 0.002 |
| History of stroke | 61 | (6.5) | 6 | (9.7) | 0.30 |
| Chronic renal failure | 41 | (4.4) | 3 | (4.8) | 0.75 |
| Cirrhosis | 7 | (0.7) | 0 | (0.0) | >0.999 |
| Cancer | 60 | (6.4) | 4 | (6.5) | >0.999 |
| History of pneumonia | 38 | (4.0) | 5 | (8.1) | 0.18 |
| History of meningitis | 8 | (0.9) | 0 | (0.0) | >0.999 |
| History of chronic sinusitis | 20 | (2.1) | 7 | (11.3) | 0.001 |
| Institution dwelling | 7 | (0.7) | 1 | (1.6) | 0.40 |
| History of influenza vaccination | | | | | <0.001 |
| Current season IV | 435 | (46.2) | 45 | (72.6) | |
| IV > 1 year | 160 | (17.0) | 8 | (12.9) | |
| No history of IV | 345 | (36.7) | 9 | (14.5) | |
| Missing data | 1 | (0.1) | 0 | (0.0) | |
| At risk of invasive pneumococcal infection | 224 | (23.8) | 21 | (33.9) | 0.07 |
| Hospitalization | | | | | 0.06 |
| Yes | 501 | (53.2) | 27 | (43.6) | |
| No | 438 | (46.6) | 34 | (54.8) | |
| Missing data | 2 | (0.2) | 1 | (1.6) | |

*Abbreviations: IV = influenza vaccination.*

** Information on self-reported pneumococcal vaccination was available for 1,003 patients. Values for the primary outcome were missing for 87 patients who died within 6 months, 275 who were lost to follow-up at 6 months, and 110 who did not answer.*

Table S6. Adjusted odds ratio of self-reported pneumococcal and influenza vaccination associated with intervention arm in intention-to-treat analysis; IMPROVED trial (N=1,475)

| Outcome | Adjusted Odds Ratio * (95% CI) | | *P-Value* |
|---|---|---|---|
| Self-reported 6-month pneumococcal vaccination | 1.28 | (0.72 to 2.25) | 0.40 |
| Self-reported 6-month influenza vaccination | 1.73 | (1.16 to 2.58) | 0.007 |

*Abbreviations: CI = confidence interval.*

** Adjusted odds ratio point estimates along with 95% confidence interval were derived from multivariable logistic regression for dependent binary variable with study arm entered as independent variable. To account for patient clustering within ED, we used random intercept model. Odds ratios were adjusted for baseline ED (location in Paris and suburbs, university affiliation, annual volume* ≥40,000) and patient (age, gender, enrollment between November 1st and April 30th, reason for ED visit, underlying conditions, history of pneumonia, *history of chronic sinusitis, history of meningitis, institution dwelling, history of influenza vaccination, and hospital admission) characteristics. Missing values were replaced by multiple imputation.*

Table S7. Comparison of baseline patient characteristics according to self-reported 6-month influenza vaccination; IMPROVED trial.

| | Self-reported 6-month influenza vaccination* | | | | |
|---|---|---|---|---|---|
| | No (*n*=486) *n or med (% or IQR)* | | Yes (*n*=387) *n or med (% or IQR)* | | *P-value* |
| Age (year) | 74 | (68–80) | 76 | (70–83) | <0.001 |
| Gender (female) | 224 | (46.1) | 208 | (53.7) | 0.03 |
| Study site | | | | | |
| Location in Paris and suburbs | 155 | (31.9) | 128 | (33.1) | 0.71 |
| University hospital | 252 | (51.9) | 223 | (57.6) | 0.09 |
| ED annual volume ≥40,000 | 271 | (55.8) | 232 | (59.9) | 0.21 |
| Enrollment between November and April | 344 | (70.8) | 192 | (49.6) | <0.001 |
| Reason for ED visit | | | | | 0.13 |
| Respiratory tract infection | 33 | (6.8) | 20 | (5.2) | |
| Non-respiratory tract infection | 11 | (2.3) | 8 | (2.1) | |
| Medical reason | 291 | (59.9) | 247 | (63.8) | |
| Traumatic reason | 100 | (20.6) | 89 | (23.0) | |
| Non-traumatic surgery | 10 | (2.1) | 6 | (1.6) | |
| Psychiatry | 1 | (0.2) | 2 | (0.5) | |
| Other | 40 | (8.2) | 15 | (3.9) | |
| At least one underlying condition | 129 | (26.5) | 119 | (30.7) | 0.17 |
| Chronic respiratory disease | 24 | (4.9) | 21 | (5.4) | 0.75 |
| Heart failure | 47 | (9.7) | 47 | (12.1) | 0.24 |
| Immunosuppression | 4 | (0.8) | 6 | (1.6) | 0.35 |
| History of stroke | 22 | (4.5) | 33 | (8.5) | 0.02 |
| Chronic renal failure | 24 | (4.9) | 17 | (4.4) | 0.71 |
| Cirrhosis | 4 | (0.8) | 3 | (0.8) | >0.999 |
| Cancer | 35 | (7.2) | 19 | (4.9) | 0.16 |
| History of pneumonia | 17 | (3.5) | 17 | (4.4) | 0.50 |
| History of meningitis | 5 | (1.0) | 2 | (0.5) | 0.47 |
| History of chronic sinusitis | 12 | (2.5) | 12 | (3.1) | 0.87 |
| Institution dwelling | 5 | (1.0) | 4 | (1.0) | >0.999 |
| History of influenza vaccination | | | | | <0.001 |
| Current season IV | 102 | (21.0) | 320 | (82.7) | |
| IV > 1 year | 103 | (21.2) | 47 | (12.1) | |
| No history of IV | 281 | (57.8) | 19 | (4.9) | |
| Missing data | 0 | (0.0) | 1 | (0.3) | |
| At risk of invasive pneumococcal infection | 118 | (24.3) | 96 | (24.8) | 0.86 |
| Hospitalization | | | | | 0.82 |
| yes | 224 | (46.1) | 185 | (47.8) | |
| No | 261 | (53.7) | 201 | (51.9) | |
| Missing data | 1 | (0.2) | 1 | (0.3) | |

Abbreviations: IV = influenza vaccination.

* The secondary outcome of self-reported influenza vaccination was available in 873 patients. Values for the primary outcome were missing for 87 patients who died within 6 months, 275 who were lost to follow-up at 6 months, and 240 who did not answer.
